# Severe Cardiovascular Collapse Associated With Remimazolam-Based Anesthetic Induction: A Case Series of Three Elderly Patients

**DOI:** 10.7759/cureus.112079

**Published:** 2026-07-05

**Authors:** Donghyo Kim, Eunju Kim

**Affiliations:** 1 Anesthesiology, Daegu Fatima Hospital, Daegu, KOR

**Keywords:** anaphylaxis, anesthesia induction, cardiac arrest, cardiovascular collapse, elderly patients, end-tidal carbon dioxide, perioperative hypersensitivity, remimazolam

## Abstract

Remimazolam is an ultra-short-acting benzodiazepine that is increasingly used for the induction and maintenance of general anesthesia owing to its favorable hemodynamic profile. However, severe perioperative hypersensitivity reactions associated with its use have been reported. We report three cases of elderly patients who experienced severe cardiovascular collapse immediately after anesthetic induction with remimazolam. Additionally, an institutional review conducted over an 18-month study period identified six suspected perioperative hypersensitivity reactions. All six patients were elderly and developed sudden cardiovascular or respiratory instability shortly after induction of anesthesia with remimazolam and rocuronium. Of these, three patients experienced severe cardiovascular collapse requiring epinephrine administration or cardiopulmonary resuscitation, and two progressed to cardiac arrest. Cutaneous manifestations were absent or minimal in several cases, whereas a marked reduction in end-tidal CO₂ and refractory hypotension were the predominant clinical features. In selected cases, elevated histamine or tryptase levels provided evidence of mast-cell activation.

Although rocuronium remains an important alternative causative agent, the temporal relationship with remimazolam administration, the recurrence pattern within the institution, and the uneventful subsequent anesthesia with alternative regimens in some patients suggest that remimazolam or its formulation components should be considered among the potential triggers. These cases highlight the need for early recognition of perioperative hypersensitivity when abrupt cardiovascular collapse and a sudden decrease in end-tidal CO₂ occur during induction, even in the absence of skin findings.

## Introduction

Remimazolam is generally considered safe based on clinical studies of general anesthesia [1]. However, cases of remimazolam-associated anaphylaxis have been reported during anesthetic induction [2]. Perioperative anaphylaxis is a rare but life-threatening event, most commonly associated with neuromuscular blocking agents or antibiotics. The incidence of perioperative anaphylaxis has been estimated to range from approximately one in 10,000 to one in 20,000 anesthetic procedures, with a reported mortality rate of 3-9% [3]. However, emerging evidence indicates that anesthetic agents themselves, including remimazolam, may also act as potential triggers of such reactions. We present three severe cases of cardiovascular collapse associated with remimazolam-based anesthetic induction and further describe institutional observations of six suspected perioperative hypersensitivity reactions identified during the study period.

We retrospectively reviewed anesthesia records at Daegu Fatima Hospital between May 2, 2024, and September 16, 2025, to identify suspected perioperative hypersensitivity reactions occurring during anesthetic induction. Suspected perioperative hypersensitivity reactions were identified through a review of anesthesia records, intraoperative adverse event reports, and serum tryptase and histamine testing data, followed by detailed chart review to confirm clinical compatibility with perioperative hypersensitivity. Six patients with suspected perioperative hypersensitivity reactions were identified among patients receiving remimazolam-based induction during the study period. Three patients who developed severe cardiovascular collapse requiring epinephrine administration or cardiopulmonary resuscitation were selected for detailed presentation. The primary objective of this report was to describe the clinical characteristics of these three most severe cases, whereas the remaining three suspected cases were included solely to provide institutional context regarding the recurrence pattern.

These patients represented the most severe cases, characterized by profound cardiovascular collapse, cardiac arrest, or ICU-level management. The remaining three patients experienced less severe reactions and were not described individually. Among these additional patients, two required epinephrine administration and demonstrated elevated serum histamine and tryptase concentrations, whereas one recovered without epinephrine treatment. Severe hypersensitivity was defined as an abrupt cardiovascular collapse temporally associated with anesthetic induction that required vasopressors, epinephrine, or cardiopulmonary resuscitation. Demographic characteristics, anesthetic medications, laboratory findings, intraoperative hemodynamic variables, treatments, and clinical outcomes were reviewed from electronic medical records. For institutional context, all patients who underwent general anesthesia at our institution during the study period (May 2, 2024, to September 16, 2025) were identified using the institutional anesthesia database.

Patients were categorized according to the induction regimen (remimazolam-rocuronium or propofol-rocuronium), and the incidence of suspected perioperative hypersensitivity reactions was summarized for each group to provide institutional safety context. No formal statistical comparison was performed because anesthetic selection was based on patient age rather than randomization. These incidence estimates were intended solely to provide institutional observational safety data and were not intended to infer causality. At our institution, remimazolam was administered to elderly patients aged 65 years or older because of its favorable hemodynamic profile, whereas propofol was used for anesthetic induction in patients younger than 65 years. Therefore, the two induction groups were not directly comparable with respect to baseline demographic characteristics.

Serum tryptase and histamine samples were collected according to institutional protocols in all three presented cases and in two additional patients who required epinephrine treatment for suspected hypersensitivity reactions. This retrospective case series and institutional database review was approved by the Institutional Review Board of Daegu Fatima Hospital (Approval No. DFH IRB 2026-06-007). The requirement for informed consent was waived owing to the retrospective design of the study and the use of anonymized clinical data.

## Case presentation

The clinical characteristics of the three patients are summarized in Table 1. The individual cases are described below.

**Table 1:** Clinical characteristics and perioperative features of the three cases ^*^Total cumulative epinephrine dose, including continuous infusion when applicable. ^**^Ring and Messmer grades III and IV indicate severe perioperative hypersensitivity reactions with life-threatening cardiovascular manifestations, whereas Grade IV includes cardiac arrest requiring cardiopulmonary resuscitation CPR: cardiopulmonary resuscitation; SBP: systolic blood pressure; EtCO₂: end-tidal carbon dioxide; PIP: peak inspiratory pressure; ICU: intensive care unit; ASA: American Society of Anesthesiologists

Clinical characteristics and perioperative features			
Age (years)	71	73	72
Sex	Male	Male	Male
Body weight (kg)	57	78	56
Previous remimazolam exposure	Yes	No	No
Hypotension	Yes	Yes	Yes
Cardiac arrest	No	Yes	Yes
CPR performed	No	Yes	Yes
Number of CPR cycles	0	4	3
Peak tryptase (μg/L)	16.7	16	5.1
Histamine (ng/mL)	7.13	16.1	14.2
Surgery type	Urologic surgery	Hernia repair	General surgery
Remimazolam dose (mg/kg)	0.28	0.21	0.18
Rocuronium dose (mg/kg)	0.61	0.51	0.89
Total epinephrine use^*^ (mg)	3.2	4	3
Lowest SBP (mmHg)	52	38	40
Lowest ETCO₂ (mmHg)	16	20	N/A
Peak PIP (cmH₂O)	28	26	26
Ring and Messmer classification^**^	III	IV	IV
ICU admission	Yes	Yes	Yes
ASA class	III	III	II
Rash/cutaneous sign	No	No	No
Time to onset (min)	5	9	8
Emergency surgery	No	No	Yes

Case 1

A 71-year-old male with benign prostatic hyperplasia and atrial fibrillation, without any known allergy history, underwent surgery under general anesthesia. He had previously received remimazolam without complications. Anesthesia was induced with remimazolam 16 mg (0.28 mg/kg) and rocuronium 35 mg (0.61 mg/kg). Shortly after induction, the patient developed severe hypotension, with systolic blood pressure decreasing to approximately 50 mmHg. Peak inspiratory pressure increased to 28 cmH₂O, and end-tidal CO₂ decreased to 16-18 mmHg.

**Figure 1:**
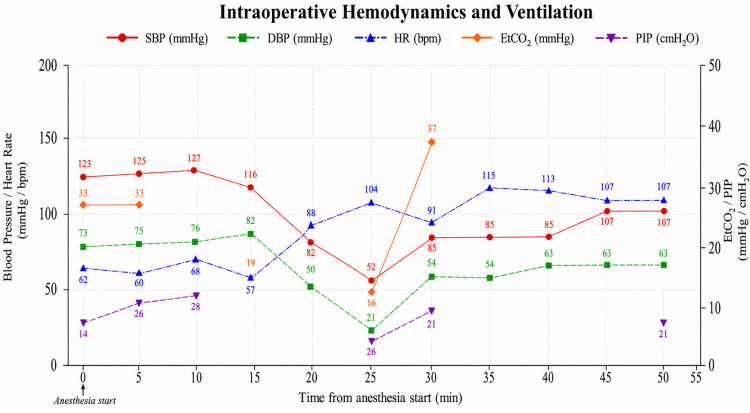
Intraoperative hemodynamic and ventilatory changes in Case 1 SBP: systolic blood pressure; DBP: diastolic blood pressure; HR: heart rate; EtCO₂: end-tidal carbon dioxide; PIP: peak inspiratory pressure

Administration of phenylephrine produced no significant response. Epinephrine boluses of 0.1 mg, 0.2 mg, and 0.5 mg were administered, after which the hemodynamic parameters stabilized. Continuous epinephrine infusion was subsequently maintained using an infusion pump during transfer. Dexamethasone and chlorpheniramine were also administered. Laboratory findings revealed elevated tryptase (16.7 μg/L) and histamine (7.13 ng/mL), supporting a diagnosis of a mast cell-mediated hypersensitivity reaction. Sugammadex was administered to reverse neuromuscular blockade. Intraoperative hemodynamic and ventilatory changes are shown in Figure 1. As illustrated in Figure 1, the onset of hypotension was accompanied by a marked decrease in end-tidal CO₂ and compensatory tachycardia. A simultaneous increase in peak inspiratory pressure suggested the possibility of bronchospasm or increased airway resistance during the reaction.

Case 2

A 73-year-old male with hypertension and no known allergy history underwent hernia repair surgery under general anesthesia using a laryngeal mask airway. Following induction with remimazolam 16 mg (0.21 mg/kg) and rocuronium 40 mg (0.51 mg/kg), the peak inspiratory pressure measured immediately after induction with the laryngeal mask airway was 14 cmH₂O. The patient subsequently developed profound hypotension (blood pressure 38/22 mmHg), accompanied by bradycardia and decreased end-tidal CO₂. Peak inspiratory pressure increased to 26 cmH₂O; therefore, the laryngeal mask airway was removed, and tracheal intubation was performed. Electrocardiography demonstrated QRS widening, followed by pulseless electrical activity.

Immediate cardiopulmonary resuscitation was initiated, and epinephrine was administered on three occasions. Dexamethasone and chlorpheniramine were also administered. Return of spontaneous circulation was achieved after approximately nine minutes, and the vital signs stabilized. After hemodynamic stabilization, surgery was continued. The patient was transferred to the ICU postoperatively, where laboratory evaluation performed after ICU admission demonstrated thrombocytopenia, prolonged coagulation parameters, and elevated D-dimer levels consistent with disseminated intravascular coagulation. Laboratory evaluation also showed markedly elevated histamine (16.10 ng/mL) and tryptase (16 μg/L), consistent with anaphylaxis.

**Figure 2:**
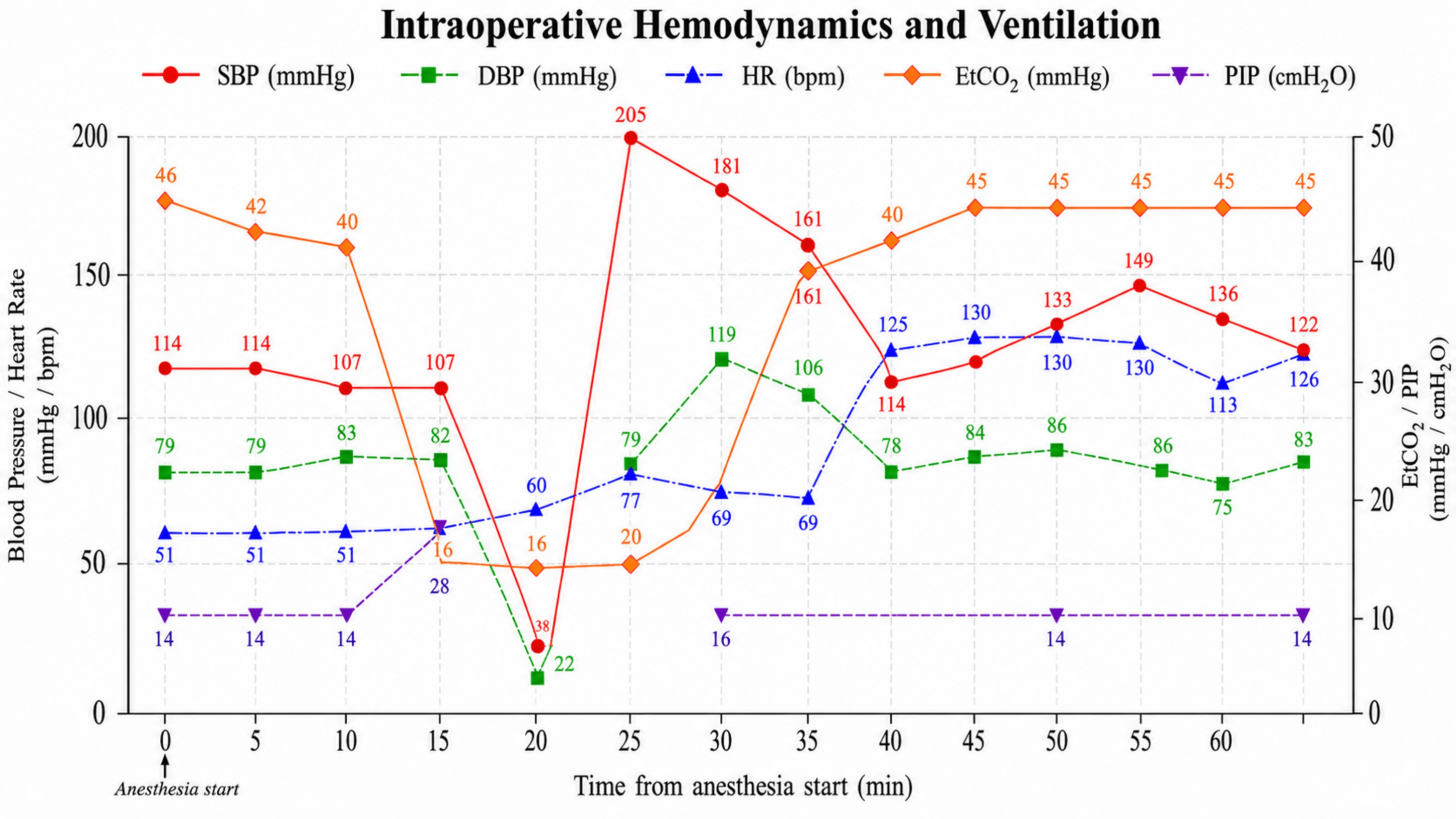
Intraoperative hemodynamic and ventilatory changes in Case 2 SBP: systolic blood pressure; DBP: diastolic blood pressure; HR: heart rate; EtCO₂: end-tidal carbon dioxide; PIP: peak inspiratory pressure

Intraoperative hemodynamic and ventilatory changes are shown in Figure 2. Figure 2 demonstrates an abrupt cardiovascular collapse characterized by profound hypotension and a marked reduction in end-tidal CO₂, followed by cardiac arrest requiring cardiopulmonary resuscitation.

Case 3

A 72-year-old male with no underlying disease or known allergy history underwent emergency surgery. After induction with remimazolam 10 mg (0.18 mg/kg) and rocuronium 50 mg (0.89 mg/kg), the peak inspiratory pressure measured immediately after induction was 12 cmH₂O and gradually increased to 26 cmH₂O. Approximately eight minutes after induction, the patient developed hypotension (55/35 mmHg), and electrocardiography showed QRS widening and ST-segment elevation. Epinephrine 1 mg (1:10,000, 10 mL) was administered, and chest compressions were initiated. Two minutes later, two additional 1-mg doses of epinephrine were administered. Dexamethasone and chlorpheniramine were also administered. During chest compressions, polymorphic ventricular tachycardia was observed, and defibrillation was being prepared; however, the rhythm converted to sinus rhythm and return of spontaneous circulation was achieved.

**Figure 3:**
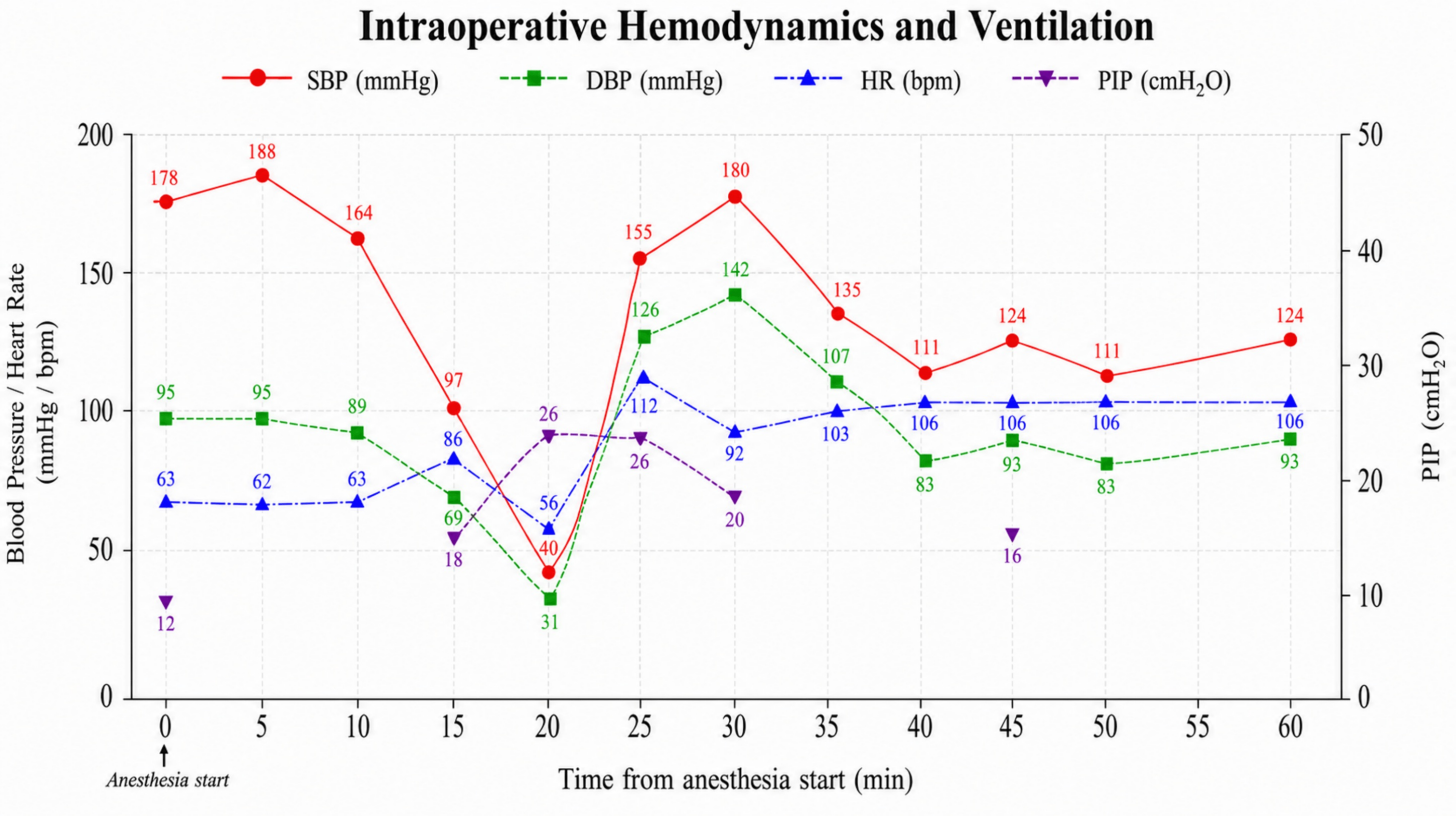
Intraoperative hemodynamic and ventilatory changes in Case 3 SBP: systolic blood pressure; DBP: diastolic blood pressure; HR: heart rate; EtCO₂: end-tidal carbon dioxide; PIP: peak inspiratory pressure

The patient regained spontaneous respiration and was extubated. Surgery was aborted, and he was transferred to the ICU. Laboratory evaluation demonstrated a serum tryptase level of 5.1 μg/L and an elevated histamine level of 14.20 ng/mL, whereas total IgE was within the normal range. Seven days later, the previously aborted surgery was reattempted. Anesthesia was induced with propofol 80 mg and rocuronium 30 mg without any adverse reaction, and anesthesia induction, maintenance, emergence, and completion of surgery were uneventful. Intraoperative hemodynamic and ventilatory changes are shown in Figure 3. As shown in Figure 3, profound hypotension rapidly developed after induction, accompanied by electrocardiographic instability and subsequent cardiac arrest. The gradual increase in peak inspiratory pressure suggested concomitant increased airway resistance during the reaction.

## Discussion

In this report, we describe three severe cases chosen from six suspected perioperative hypersensitivity reactions identified during an institutional review. All cases were characterized by rapid cardiovascular collapse, with two patients experiencing cardiac arrest requiring cardiopulmonary resuscitation, reflecting the life-threatening severity of these reactions. The severity of each reaction was classified according to the Ring and Messmer grading system [4]. Based on this classification, all three patients met the criteria for severe perioperative hypersensitivity, with Cases 2 and 3 classified as Grade IV reactions due to cardiac arrest requiring cardiopulmonary resuscitation.

A key finding of this case series is the consistent temporal association between remimazolam-based induction and the onset of symptoms. Perioperative anaphylaxis is most commonly associated with neuromuscular blocking agents and antibiotics. At our institution, remimazolam has been administered preferentially to patients aged 65 years or older for anesthetic induction due to its favorable hemodynamic profile. During the 18-month study period, six patients developed suspected perioperative hypersensitivity reactions, all of whom had received remimazolam during induction. Although this observational finding cannot establish causality, the exclusive occurrence of suspected reactions in elderly patients receiving remimazolam raises concern regarding a potential association. Interestingly, all three patients in the present series were older than 70 years and developed predominantly cardiovascular manifestations with minimal or absent cutaneous findings. Previous studies have suggested that perioperative anaphylaxis in older adults may present atypically and may be associated with more severe cardiovascular compromise [5].

Elevated tryptase and histamine levels in Cases 1-3 provided biochemical evidence of mast cell activation, consistent with anaphylaxis. Elevated levels of these mediators were also observed in two additional patients who required epinephrine treatment for suspected hypersensitivity reactions, further supporting that mast cell activation was not confined to the three severe cases described in this report. Measurement of serum tryptase is recommended as a reliable biomarker for confirming mast cell activation in suspected perioperative anaphylaxis [6]. However, an elevated tryptase level does not distinguish between IgE-mediated and non-IgE-mediated mechanisms. Case 3 demonstrated a relatively lower tryptase level, which may reflect variability in mediator release or differences in the timing of sample collection. Importantly, normal or only mildly elevated tryptase levels do not exclude perioperative anaphylaxis, particularly when sampling timing is suboptimal or when mediator release is transient.

Furthermore, serum tryptase sensitivity is imperfect and may be reduced when blood sampling is delayed or when reactions predominantly manifest as cardiovascular collapse without prominent cutaneous findings. Therefore, the relatively low tryptase level in Case 3 should be interpreted cautiously in the context of the clinical presentation, including abrupt cardiovascular collapse and the need for resuscitative treatment. Although serum tryptase elevation was modest in Case 3, the elevated histamine level supports mast cell mediator release and provides additional biochemical evidence for a hypersensitivity reaction. Current international guidelines emphasize that normal or only mildly elevated serum tryptase concentrations do not exclude perioperative anaphylaxis, particularly when sampling is delayed or when reactions predominantly manifest as cardiovascular collapse [6,7]. Furthermore, the abrupt onset immediately following induction, characteristic cardiovascular collapse, electrocardiographic abnormalities, and favorable response to epinephrine support the clinical diagnosis of perioperative hypersensitivity.

The clinical patterns observed suggest the possibility of both IgE-mediated and non-IgE-mediated mechanisms [8,9]. Case 1 developed a severe reaction upon re-exposure after a previously uneventful administration, supporting a potential IgE-mediated mechanism involving prior sensitization. In contrast, Cases 2 and 3 occurred during first known exposure, suggesting the possibility of non-IgE-mediated hypersensitivity. However, prior sensitization cannot be completely excluded due to potential cross-reactivity or unrecognized exposure.

Recent attention has focused on dextran 40, an excipient contained in remimazolam formulations, as a potential contributor to remimazolam-associated hypersensitivity reactions. Recent evidence suggests that non-IgE-mediated pathways involving complement activation may contribute to hypersensitivity reactions and mast cell activation, resulting in severe cardiovascular manifestations [8]. Several recent case reports have suggested a possible role for dextran 40 in remimazolam-associated hypersensitivity [2,10-12]. In some cases, skin testing demonstrated positive responses to dextran 40 despite negative or inconclusive results for remimazolam itself, raising the possibility that formulation components rather than the active drug may contribute to certain reactions [10,11]. However, the currently available evidence remains limited to case reports and small case series, and the relative contribution of remimazolam and dextran 40 has not yet been clearly established [12]. Therefore, the potential role of dextran 40 requires further mechanistic and clinical investigation.

Neuromuscular blocking agents are among the most frequently implicated causes of perioperative anaphylaxis, and rocuronium remains an important differential consideration [3,7]. Therefore, despite the temporal association observed in the present series, the possibility that rocuronium contributed to the reactions cannot be excluded. Although rocuronium remains an important alternative explanation, the clustering of six suspected hypersensitivity reactions during remimazolam-based induction within a relatively short period and the growing number of published reports raise concerns that remimazolam or its formulation components may have contributed to these events. Nevertheless, the reproducible temporal relationship between remimazolam administration and symptom onset across multiple cases raises suspicion of a possible association.

Notably, Case 3 subsequently underwent repeat surgery seven days later using propofol and rocuronium without recurrence of hypersensitivity. Although this observation does not establish causality or definitively exclude rocuronium as a contributing factor, successful re-exposure to rocuronium without recurrence in Case 3 makes rocuronium alone a less likely sole explanation, although it does not definitively exclude rocuronium as a contributing factor [7,13]. Causality cannot be definitively established without confirmatory allergy testing, such as skin testing, basophil activation testing, specific IgE assays, or drug provocation testing. Importantly, remimazolam-associated hypersensitivity may occur without typical cutaneous manifestations, which can delay recognition.

Review of the intraoperative trends shown in Figures 1-3 revealed a consistent pattern of abrupt hypotension accompanied by a decrease in end-tidal CO₂ shortly after remimazolam administration. In all three cases, a marked decline in end-tidal CO₂ preceded or accompanied severe hypotension during cardiovascular collapse. Notably, cutaneous manifestations were absent or not readily apparent, emphasizing the importance of hemodynamic and ventilatory monitoring for early recognition of perioperative hypersensitivity reactions. Accordingly, a sudden decrease in end-tidal CO₂ during anesthetic induction, particularly when accompanied by refractory hypotension, should prompt consideration of perioperative hypersensitivity even in the absence of cutaneous manifestations [6,7]. The marked decrease in end-tidal CO₂ likely reflected severely impaired pulmonary perfusion secondary to profound circulatory collapse, a well-recognized physiologic consequence of markedly reduced cardiac output during severe anaphylaxis [7].

Although Case 1 appeared clinically less severe than Cases 2 and 3, the total epinephrine requirement was greater because continuous epinephrine infusion was maintained after initial stabilization. Therefore, the epinephrine dose in Case 1 should be interpreted in the context of ongoing infusion rather than as reflecting more severe cardiovascular collapse than the other two cases. Case 2 also raises an important clinical consideration. Severe anaphylaxis can be complicated by disseminated intravascular coagulation, and postoperative laboratory findings in this patient were consistent with this pattern. Even when vital signs appear stable after resuscitation, ongoing surgery may impose additional physiologic stress and contribute to cellular injury after a severe systemic reaction. In retrospect, this case suggests that postponement of non-emergent surgery may be appropriate after severe perioperative hypersensitivity, even when apparent hemodynamic stabilization has been achieved.

The institutional incidence of perioperative hypersensitivity reactions according to induction regimen is summarized in Table 2.

**Table 2:** Institutional incidence of perioperative hypersensitivity reactions according to induction regimen

Variable	Propofol–rocuronium	Remimazolam–rocuronium
Total patients	3,665	2,415
Hypersensitivity reactions, n	0	6
Incidence (%)	0	0.25

During the same 18-month institutional review period, six suspected perioperative hypersensitivity reactions were identified among 2,415 patients receiving remimazolam-rocuronium induction, whereas no suspected reactions were identified among 3,665 patients receiving propofol-rocuronium induction, corresponding to an incidence of 0.25% and 0%, respectively. Because anesthetic selection at our institution was determined by patient age rather than random allocation, direct statistical comparison between the two induction regimens was inappropriate. Therefore, these observations are presented solely as an institutional observational safety signal rather than evidence of a causal relationship or treatment effect. The occurrence of six suspected hypersensitivity reactions within a single institution over 18 months raises concern that remimazolam-associated hypersensitivity may be underrecognized in routine clinical practice, particularly because cardiovascular manifestations may occur without obvious cutaneous signs [7,12]. Although these observations are hypothesis-generating, they warrant further investigation in larger, controlled studies.

Certain drugs may induce non-IgE-mediated reactions through histamine release and complement activation, potentially resulting in significant hemodynamic instability [9]. However, unlike classical dose-dependent pharmacologic effects, such reactions may occur even at standard or low doses, particularly in susceptible individuals. Evidence from other intravenous agents, such as vancomycin-associated infusion reactions, suggests that rapid administration may enhance non-IgE-mediated histamine release and increase the severity of hypersensitivity-like reactions [14]. However, direct evidence linking infusion rate to remimazolam-associated hypersensitivity is currently lacking, and this hypothesis remains extrapolative.

These cases highlight several important clinical implications. First, remimazolam, despite its favorable hemodynamic profile, may be associated with severe hypersensitivity reactions. Remimazolam is known for its rapid onset and recovery profile, along with minimal hemodynamic instability compared with conventional agents such as propofol [1]. Second, prior uneventful exposure does not exclude the possibility of subsequent IgE-mediated anaphylaxis. Third, life-threatening anaphylactoid reactions may occur even during first known exposure because non-IgE-mediated mechanisms do not require prior sensitization, although unrecognized sensitization cannot be completely excluded [8,9].

Notably, all three patients were elderly males and developed severe cardiovascular collapse within minutes of induction. Although definitive risk factors cannot be identified from this small series, clinicians should remain vigilant for severe hypersensitivity reactions during remimazolam administration, particularly when abrupt cardiovascular collapse occurs during anesthetic induction. Early recognition of declining end-tidal CO₂ may facilitate more rapid diagnosis and treatment of severe perioperative hypersensitivity.

The lack of confirmatory diagnostic testing, such as skin testing, basophil activation testing, specific IgE assays, or drug provocation testing, represents a major limitation of this report and precludes definitive determination of causality. Current perioperative hypersensitivity guidelines recommend systematic postoperative allergy evaluation, including skin testing and, when appropriate, basophil activation testing, to improve diagnostic certainty and facilitate future anesthetic planning [7].

Because the reactions occurred unexpectedly and the patients were not routinely referred for formal postoperative allergy evaluation, confirmatory testing was unavailable. In addition, the absence of serial tryptase measurements may limit the accuracy of biochemical interpretation. Although confirmatory testing was not performed, the consistent temporal relationship and reproducibility across multiple cases support a possible association with remimazolam. Therefore, these cases should be regarded as probable remimazolam-associated hypersensitivity reactions rather than definitively confirmed remimazolam-induced anaphylaxis.

The temporal association observed in our cases suggests that remimazolam may have contributed to the observed cardiovascular collapse, although causality cannot be definitively established. Given the severity of these events, anesthesiologists should maintain a high index of suspicion for hypersensitivity reactions during induction and be prepared for immediate management, including early administration of epinephrine. Epinephrine remains the first-line treatment for anaphylaxis and should be administered promptly when the condition is suspected [7].

Given the increasing number of published reports, remimazolam-associated hypersensitivity should be considered a rare but increasingly recognized perioperative complication [10-12,15]. Compared with previously reported remimazolam-associated anaphylaxis cases, which have generally been limited to isolated case reports with variable clinical severity, the present series is notable for the advanced age of all patients and the severity of cardiovascular manifestations, including cardiac arrest in two cases [2,10-12,15]. Continued pharmacovigilance and systematic reporting of suspected cases will be essential to clarify its true incidence and underlying mechanisms [12,15].

Further studies are required to elucidate the underlying immunologic mechanisms and to determine the true incidence of remimazolam-associated hypersensitivity reactions. Based on the temporal relationship observed across these cases and previously published reports, remimazolam should be considered among the potential causative agents when abrupt cardiovascular collapse occurs immediately after induction, particularly when accompanied by a marked decrease in end-tidal CO₂ [2,7,10-12,15].

## Conclusions

Remimazolam may be associated with severe hypersensitivity reactions, including life-threatening anaphylaxis, even in patients without prior exposure. Although causality could not be definitively established, remimazolam should be considered among the potential causative agents in patients who develop abrupt cardiovascular collapse immediately after anesthetic induction, particularly when accompanied by a marked decrease in end-tidal CO₂. Alternative causes, including neuromuscular blocking agents, should also be considered. Prompt recognition and immediate epinephrine administration remain essential. In the present cases, a sudden decrease in end-tidal CO₂ accompanied by refractory hypotension was a consistent feature of severe cardiovascular collapse. Further studies incorporating confirmatory allergy testing are required to clarify the underlying mechanisms and better define the relationship between remimazolam and perioperative hypersensitivity reactions. Clinicians should maintain a high index of suspicion for remimazolam-associated hypersensitivity when unexplained cardiovascular collapse occurs during anesthetic induction.
